# The effectiveness of opioid substitution treatments for patients with opioid dependence: a systematic review and multiple treatment comparison protocol

**DOI:** 10.1186/2046-4053-3-105

**Published:** 2014-09-19

**Authors:** Brittany Burns Dennis, Leen Naji, Monica Bawor, Ashley Bonner, Michael Varenbut, Jeff Daiter, Carolyn Plater, Guillaume Pare, David C Marsh, Andrew Worster, Dipika Desai, Zainab Samaan, Lehana Thabane

**Affiliations:** 1 Department of Clinical Epidemiology and Biostatistics, McMaster University, Hamilton, Canada; 2 Population Genomic Program, Chanchlani Research Centre, McMaster University, Hamilton, Canada; 3 Michael G. DeGroote School of Medicine, McMaster University, Hamilton, Canada; 4 McMaster Integrative Neuroscience Discovery & Study (MiNDS) Program, McMaster University, Hamilton, Canada; 5 Ontario Addiction Treatment Centres, Richmond Hill, Canada; 6 Northern Ontario School of Medicine, Sudbury, ON, Canada; 7 Department of Medicine, Hamilton General Hospital, Hamilton, Canada; 8 Department of Psychiatry and Behavioral Neurosciences, McMaster University, Hamilton, Canada; 9 Population Health Research Institute, Hamilton Health Sciences, Hamilton, Canada; 10 Department of Pediatrics and Anesthesia, McMaster University, Hamilton, Canada; 11 Centre for Evaluation of Medicine, St Joseph's Healthcare—Hamilton, Hamilton, Canada; 12 Biostatistics Unit, Father Sean O'Sullivan Research Centre, St Joseph's Healthcare—Hamilton, 3rd Floor, Martha Wing, Room H-325, 50 Charlton Avenue East, Hamilton, ON L8N 4A6, Canada

**Keywords:** Opioid substitution therapies, Opioid dependence, Methadone, Buprenorphine/naloxone, Naltrexone, Heroin, Systematic review, Network meta-analysis

## Abstract

**Background:**

Opioids are psychoactive analgesic drugs prescribed for pain relief and palliative care. Due to their addictive potential, effort and vigilance in controlling prescriptions is needed to avoid misuse and dependence. Despite the effort, the prevalence of opioid use disorder continues to rise. Opioid substitution therapies are commonly used to treat opioid dependence; however, there is minimal consensus as to which therapy is most effective. Available treatments include methadone, heroin, buprenorphine, as well as naltrexone. This systematic review aims to assess and compare the effect of all available opioid substitution therapies on the treatment of opioid dependence.

**Methods/Design:**

The authors will search Medline, EMBASE, PubMed, PsycINFO, Web of Science, Cochrane Library, Cochrane Clinical Trials Registry, World Health Organization International Clinical Trials Registry Platform Search Portal, and the National Institutes for Health Clinical Trials Registry. The title, abstract, and full-text screening will be completed in duplicate. When appropriate, multiple treatment comparison Bayesian meta-analytic methods will be performed to deduce summary statistics estimating the effectiveness of all opioid substitution therapies in terms of retention and response to treatment (as measured through continued opioid abuse).

**Discussion:**

Using evidence gained from this systematic review, we anticipate disseminating an objective review of the current available literature on the effectiveness of all opioid substitution therapies for the treatment of opioid use disorder. The results of this systematic review are imperative to the further enhancement of clinical practice in addiction medicine.

**Systematic review registration:**

PROSPERO CRD42013006507.

## Background

Opioids are psychoactive analgesic drugs prescribed for pain relief and palliative care [1]. Due to their addictive nature, effort and vigilance in controlling prescriptions is needed to avoid misuse and dependence. Despite such effort, opioid use disorder is commonly associated with both illicit and prescription opioid use [2]. The DSM-5 characterizes opioid use disorder as a ‘problematic pattern of opioid use leading to clinically significant impairment or distress’ [3]. Characteristics of opioid use disorder include increased tolerance, continued use despite personal and social problems, as well as withdrawal and tolerance, among other behavioral changes [3]. Opioid use has been on the rise for the past several years, although common and available treatment options have not adjusted to meet the increasing demand for therapy [4].

The rapid rise in opioid prescriptions worldwide in conjunction with the increase in misuse and addiction is concerning [2,5]. Opioid-related deaths in ON, Canada have doubled between 1991 and 2004 [6,7]. In the United States, opioid sales have surged 627% between 1997 and 2007 [8]. Accompanying this dramatic rise in prescription opioid sales, the number of opioid-related overdoses in the United States has increased tenfold since 1990 [9]. Aside from the negative impact of drug use on the patient's lifestyle and psychological state, many physical health issues are associated with opioid abuse. For instance, IV opioid use is found to be associated with serious cardiac abnormalities such as infective endocarditis [10,11]. Furthermore, opioid use has been correlated with increase HIV risk and susceptibility to other opportunistic infections such as hepatitis C and tuberculosis [12].

Today, opioid substitution treatment (OST) is used to treat opioid dependence. This medical intervention employs strategies to control rather than prevent drug use in attempts to limit the incidence of adverse events. This involves prescribing controlled amounts of longer acting but less euphoric opioids to reduce cravings and prevent withdrawal symptoms [13]. Currently, the most commonly used substitute opioid is methadone [14,15]. First introduced for the treatment of opioid use disorder in 1965, methadone maintenance treatment (MMT) has been shown to be effective in ameliorating symptoms of opioid craving and reducing the negative effects that illicit drug use has on individuals, such as increased HIV risk [16]. It has also been shown to alleviate some of the burden that illicit drug use places on society, including criminal acts and the spread of infectious disease to others [15,17-21]. Reported methadone effectiveness varies by studies, with some investigations reporting as low as 20% to as high as 70% [10-12]. These rates are largely accounted for by the numerous definitions of methadone effectiveness reported in the literature. Interindividual variability in clinical responses to methadone and dose requirements depend on several factors including age, diet, metabolism, protein binding, medications, genetic variants, and other substance use [22-26].

MMT is used by 20%–25% of opioid-dependent individuals in North America, leaving approximately 75% of the opioid-dependent population on another intervention or without any treatment at all [27]. While methadone is claimed to be an effective treatment for patients with opioid use disorder, it is important to note that alternative therapies are on the rise. Suboxone**®** is a relatively new drug approved in Canada since 2007, comprised of a combination of buprenorphine and naloxone in a 4:1 ratio [28]. When taken sublingually, only buprenorphine exerts its partial agonistic effects because naloxone is not adequately absorbed. However, in case of parenteral abuse, the administration naloxone exerts a withdrawal effect in opioid-dependent patients [29,30]. Therefore, the role of this combination is to ultimately alleviate withdrawal symptoms while also deterring intravenous use of the medication. Suboxone's effects are less prominent than full opioid agonists, as such it induces less physical dependence than other full opioid agonists such as heroin, morphine, and methadone [31]. It is also associated with less dysphoric effects than methadone, encouraging a greater portion of patients to continue in treatment. As well, it has a ceiling effect, such that its effectiveness remains constant beyond a certain dose, thus helping to control use and limit abuse [32].

According to one study, buprenorphine/naloxone patients reported significantly improved social life, educational level, and response to treatment (measured through urine toxicology screens), as compared to patients on MMT [33]. However, further studies including a 17-week randomized single-center trial reported no significant difference in the proportion of opioid-negative urine samples between patients on buprenorphine relative to methadone [31].

Naltrexone, another alternative opioid substitution therapy, is a competitive opioid receptor antagonist that blocks the euphoric effects of opioids by acting on receptors in the brain [34]. The oral form has been available since 1980s but due to the lack of patients' adherence to the therapy, it has been deemed ineffective until the recent introduction of long-acting injections and implants of naltrexone [34]. Long-lasting injectable naltrexone therapy was approved by the FDA in 2010 after a 6-month placebo-controlled trial showed that over 50% of patients remained on treatment and refrained from using illicit drugs for the entire study period [34,35]. A double-blinded randomized controlled trial (RCT) (*n* = 60) investigating the efficacy of injectable naltrexone against placebo demonstrated a significantly reduced ‘need’ for heroin, as per patient reporting, after treatment (192 or 384 mg) in comparison to placebo [36]. Naltrexone is easy to administer, does not induce tolerance over time, and it is not addictive [37].

However, naltrexone removes tolerance to opioids and thus increases the risk of overdose should patients choose to abstain from therapy and return to illicit opioid use. According to a search of the National Coronial Information System (2000–2003), deaths associated with oral naltrexone use are three to seven times higher than those of methadone [38].

Heroin-assisted therapy (HAT) is a novel and controversial treatment for opioid dependence which involves the administration of injectable diacetylmorphine, the active ingredient of heroin. HAT is more effective than oral methadone in terms of both reduction of illicit drug use (67.0% and 47.7%) and increase in retention in treatment (87.8% vs 54.1%). A study by Oviedo-Joekes et al. has shown that HAT is slightly more effective than methadone for increasing quality of life years gained (7.46 vs 7.92) [39]. As well, the study shows that HAT is more cost effective in terms of long-term incurred societal costs compared to methadone, primarily due to the fact that patients adhere to treatment longer and are less likely to relapse, resulting in less criminal activity [40].

Due to the aforementioned concerns and inconsistent findings related to the effectiveness of OSTs currently available, it is important to determine the most effective OST for increasing patient retention and restraining illicit opioid use. This systematic review will investigate the effectiveness of methadone, buprenorphine/naloxone, naltrexone, heroin-assisted therapy (HAT) and any other OST in terms of the continued opioid use (response to treatment) retention in treatment, physical and psychological well-being, social implication (criminal activity), as well as incidence of adverse events or toxic effects from the opioid intervention.

### Objectives

This systematic review aims to assess and compare the effectiveness of all available OSTs in the treatment of opioid use disorder, including but not limited to methadone, buprenorphine/naloxone (Suboxone®), naltrexone, and heroin (diacetylmorphine)-assisted therapy. Specifically, the objectives of this investigation include:

1) Assessing the effectiveness of the aforementioned therapies based on retention in treatment and continued opioid use (response to treatment).

2) Conduct direct comparisons using random effects meta-analytic models and when appropriate, conduct a network meta-analysis to synthesize a mean difference, relative risk, or odds ratio that encompasses results from multiple studies.

3) Critically evaluate current literature and identify important areas of addiction medicine that future research should address.

4) Offer unbiased report of the effectiveness of different treatments in relation to one another to enhance current clinical treatment of opioid use disorders.

### Research question

Among patients being treated for opioid use disorder, which OST (methadone, buprenorphine/naloxone, naltrexone, HAT, and/or other) is most effective for increasing retention in treatment and restraining continued opioid use?

## Methods/Design

### Data sources and search strategy

In order to conduct a comprehensive search of the available literature, we will use a set of predetermined and separate key terms to search the following online databases: Medline, EMBASE, PubMed, PsycINFO, Web of Science, Cochrane Library, Cochrane Clinical Trials Registry, World Health Organization (WHO) International Clinical Trials Registry Platform Search Portal, and the National Institutes for Health (NIH) Clinical Trials Registry. Searches will be performed independently by two authors (LN and BD). The authors will perform additional manual searches of all completed Cochrane reviews examining the effect of different OSTs. The manual search will be used to identify any RCT or observational study on OSTs that have been combined statistically and narratively in a Cochrane review, as Cochrane is the leader and gold standard in systematic reviews. We will also contact each primary investigator listed on the NIHs Clinical Trial Registry from studies deemed eligible during the title screening, where we will inform the investigators of our review and ask for information regarding any publications resulting from their trial. We will also contact a librarian from the McMaster Faculty of Health Sciences Library with expertise in systematic reviews throughout the process of devising the search strategy and conducting the literature search. The two authors (LN and BD) will then independently refer to the bibliographies of articles that pass the initial abstract screening. No constraints will be set on language or date of publication in order to allow for a more thorough search of the literature. However, only human studies will be included. As well, we will eliminate incomplete studies, as they would not provide sufficient data for extraction. We will inform the authors of the eligible articles about the review during the data extraction process to consult them for clarification of their data when needed. Please refer to Table 1 for full search strategy.

**Table 1:** Defined search strategy for the extraction of pertinent studies from multiple databases

	
CINAHL search strategy search = ___	1. (MH ‘methadone+’)
	2. (MH ‘suboxone+’)
	3. (MH ‘heroin assisted treatment+’)
	4. (MH ‘diacetylmorphine+’)
	5. (MH ‘heroin adjusted therapy+’)
	6. (MH ‘buprenorphine+’)
	7. (MH ‘Revia+’)
	8. (MH ‘Depade+’)
	9. (MH ‘Naltrexone+’)
	10. 1 or 2 or 3 or 4 or 5 or 6 or 7 or 8 or 9
	11. (MH ‘disorder, substance abuse’) or (MH ‘substance withdrawal syndrome+’) or (MH ‘substitute opioid therapy+’)
	12. 10 AND 11
Medline search strategy search = ____	1. methadone/th [Therapy]
	2. limit 1 to humans
	3. opioid substitution treatment/ae mo [adverse effects, mortality]
	4. limit 3 to humans
	5. substance-Related Disorders/de, ep, th [Drug Effects, Epidemiology, Therapy]
	6. Limit 5 to humans
	7. Opiate Substitution Treatment/or Naloxone/or Buprenorphine/or Opioid-Related Disorders/or Heroin Dependence/or Substance Withdrawal Syndrome/or Narcotic Antagonists/
	8. Limit 7 to humans
	9. Naltrexone/ae, ag, ai, tu [Adverse Effects, Agonists, Antagonists & Inhibitors, Therapeutic Use]
	10. Limit 9 to humans
	11. Substance Abuse Treatment Centers/or Substance Abuse, Intravenous/or Heroin/or Heroin Dependence/or Opioid-Related Disorders/or Randomized Controlled Trials as Topic/or Methadone/
	12. Limit 11 to humans
	13. methadone/
	14. limit 13 to humans
	15. 2 OR 4 OR 8 OR 10 OR 12 OR 14
	16. 15 AND 6
Web of science search strategy search = _____	1. Topic = (‘methadone’ OR ‘methadone maintenance therapy’ OR ‘naltrexone’ OR ‘suboxone’ OR ‘buprenorphine’ OR ‘heroin assisted treatment’)
	2. Topic = (‘substitute opioid therapy’ OR ‘opioid substitution therapy’)
	3. 1 AND 2
EMBASE search strategy Search = ___	1. methadone treatment/or methadone/or methadone plus naloxone/
	2. limit 1 to human
	3. buprenorphine plus naloxone/
	4. limit 3 to human
	5. morphine sulfate plus naltrexone/or naltrexone/
	6. limit 5 to human
	7. opiate addiction/or heroin dependence/or methadone/or diamorphine/
	8. limit 7 to human
	9. opiate substitution treatment/ae [Adverse Drug Reaction]
	10. methadone/or buprenorphine/or opiate addiction/or substitute opioid therapy.mp.
	11. 9 or 10
	12. 2 or 4 or 6 or 8
	13. 11 and 12
	14. substance abuse/or addiction/or drug dependence/
	15. 13 and 14
	16. randomized controlled trial/
	17. 15 and 16
PsycINFO search strategy search = _____	1. exp Methadone Maintenance/or exp Methadone/
	2. limit 1 to human
	3. exp Treatment Outcomes/or exp Drug Therapy/or exp Methadone Maintenance/or exp Drug Dependency/or exp Maintenance Therapy/or exp Methadone/or exp Heroin/
	4. limit 3 to human
	5. exp Drug Addiction/or exp Clinics/or exp Drug Therapy/or exp Drug Dependency/or exp ‘Recovery (Disorders)’/or exp Maintenance Therapy/
	6. limit 5 to humans
	7. exp naltrexone/
	8. limit 7 to humans
	9. exp Maintenance Therapy/or exp Naloxone/or exp Drug Therapy/or exp Drug Dependency/or exp Heroin Addiction/
	10. limit 7 to humans
	11. exp Treatment Outcomes/or exp Clinical Trials/or exp Drug Therapy/or exp Heroin Addiction/or exp Methadone Maintenance/
	12. limit 9 to humans
	13. 2 and 8 and 10
	14. 2 and 10
	15. 2 and 12
	16. 2 and 6 and 12
	17. 13 or 14 or 15 or 16
Cochrane library search strategy search = __	1. search title, abstract, keywords: methadone
	2. search title, abstract, keywords: buprenorphine
	3. search title, abstract, keywords: naltrexone
	4. search title, abstract, keywords: heroin assisted treatment
Clinical Trials Registry through National Institutes for Health search strategy Search = ____	‘methadone’ OR ‘suboxone’ OR ‘Buprenorphine’ OR ‘substitute opioid therapy’ OR ‘naltrexone’ OR ‘heroin assisted treatment’ OR ‘heroin adjustment therapy’ AND ‘opioid addiction’, with additional criteria including: Completed studies, exclude unknown status, adult age requirements, and all trials had to be listed as Phase 3, 4
Cochrane Central Register of Controlled Trials search strategy Search = ____	‘substitute opioid therapy’ OR ‘methadone’ OR ‘naltrexone’ OR ‘buprenorphine’ OR ‘heroin assisted treatment’ OR ‘heroin adjustment therapy’ in title abstract keywords and opioid addiction in title abstract keywords in Trials

### Selection of studies

**Figure 1:**
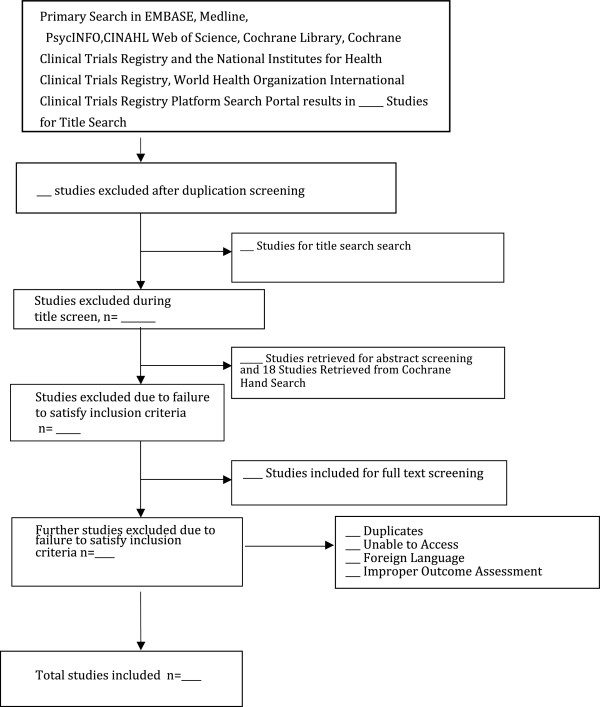
Flow diagram of screening process.

The authors (LN and BD) will independently conduct a primary title search, title screening, abstract screening, and full-text extraction. We will refer to the inclusion and exclusion criteria throughout the screening process. All data extraction forms will be pilot tested before use. In the case of a disagreement during the search and selection process, we will engage in a discussion to reach a mutual agreement. However, should the conflict persist, we will resort to a third author (ZS) to facilitate the resolution. Agreement level between reviewers will also be assessed using the kappa statistic [41]. As per guidelines set by the Meta-analysis of Observational Studies in Epidemiology (MOOSE) and the Preferred Reporting Items for Systematic Reviews and Meta-Analyses (PRISMA), we will include both a flow diagram displaying screening process (Figure 1) and a detailed table of the studies selected in the systematic review [42,43].

### Inclusion and exclusion criteria

The authors will limit the studies included in this review to RCTs and observational studies evaluating the effectiveness of methadone, buprenorphine/naloxone, naltrexone, HAT, and/or any other unlisted OST for the treatment of opioid dependence. The study will have had to examine the effectiveness of one of the aforementioned treatments with one or more of the outcomes of interest: retention in treatment, response to treatment (as measured through continued opioid use), criminal activity (as measured by self-report), mortality, physical and psychological health, as well as incidence of toxic and adverse events. No age restrictions will be set. In addition, we will also exclude articles examining specialized populations such as prisoners examined within penitentiaries or other settings as well as pregnant women. All studies must also be primary investigations with comparison groups (separated by a treatment or placebo), we will not allow studies such as case reports or case series to be included in the review, arguably due to their lack of an appropriate comparison group. We have noted that we are primarily interested in patient important long-term outcomes such as illicit substance abuse behavior, retention in treatment, and side effects; we will not be including studies whose primary objective is to determine dosing and detoxification effects or precipitated withdrawal. Our primary concern is the influence of OSTs on retention in treatment and restraining continued opioid use. We are not interested in studies determining the effectiveness of OSTs on other substances such as cocaine or alcohol. We will not include pilot studies or RCTs at phases 0, 1, and 2. We will review any studies indexed within the databases allotted time frame and no restrictions on publication date will be set. All studies selected for inclusion into the manuscript will be required to demonstrate appropriate ethics committee approval in accordance with the objectives stated within the Helsinki Declaration. This investigation will not require direct human experimentation; however, we will still comply with all objectives of the Helsinki Declaration.

### Quality assessment of individual studies

Two authors (LN and BD) will independently conduct a methodological quality assessment of the studies selected for the systematic review. We will use the Newcastle-Ottawa Scale (NOS) for observational studies to assess the risk of bias [44]. We will use the Cochrane risk of bias tool to assess the risk of bias for RCTs [45]. Discussions will be used to resolve any discrepancies that should arise. A third author (ZS) will be contacted to facilitate resolution in the case that a mutual consensus is not reached. When assessing risk of bias using the Cochrane tool for RCTs, scores of 1, 2, or 3 will be assigned for each domain that is ranked as ‘low risk’, ‘unclear’, or ‘high risk’ of bias, respectively. Scores from all the domains addressed by the Cochrane risk of bias tool will be added to give a total score out of 18, with higher scores indicating a higher risk of bias. When assessing risk of bias using the modified Newcastle Ottawa Scale for observational studies, scores of 1, 2, or 3 will be assigned for domains ranked as ‘high risk’, ‘unclear’, or ‘low risk’ of bias. Scores from all the domains will be totaled to a score of 21, with higher scores indicating lower risk of bias.

### Outcome measures

This systematic review will compare methadone, buprenorphine/naloxone, naltrexone, and HAT among other substitute opioid therapies in terms of retention and response to treatment (as measured through continued opioid use). Retention in treatment has multiple definitions and measurements across studies, where some chose to define retention as a continuous value such as the number of days a patient continued in treatment until the last day of receiving an intervention receipt [46]; other studies chose to measure retention as a binary outcome such as the percentage of patients who completed their treatment course [47]; and lastly, some studies chose to report the number of patients who received the treatment for a predefined number of treatment days [39]. Due to the numerous ways retention is defined, measured, and reported, we will collect any information the articles offer on patient retention. We will statistically combine results from studies that similarly report, measure, and define retention. We will contact authors of studies who uniquely measure/report patient retention results in an effort to obtain results in the more commonly defined retention method.

Furthermore, we define response to treatment as abstinence from use of illicit opioids as indicated by absence of any opioids not pertaining to the treatment in urine toxicology screening. We will compare the percentage of opioid-negative urine samples between treatments, calculated by dividing the number of opioid-negative urine screens by the total number of urine samples provided as used in the studies by Mattick et al. (2003) and Samaan et al. (2014) [48,49]. Please refer to Table 2 for detailed information on how these variables are defined and measured in the current literature.

**Table 2:** Definitions of outcomes in opioid substitution investigations

**Outcome**	**Definition**	**Measurement of variable (units)**	**Statistical estimates and measurement of association of this outcome**	**Studies**
Continued illicit drug abuse	Abstaining from illicit opioid use throughout treatment.	-Urine toxicology screening	OR, rate ratio	[39,46,47,50,51]
		-Self-reported drug use		
Retention in treatment	Proportion or participants completing treatment and days in treatment from beginning of the study until the last day of therapy.	-Number of days patient remains in treatment (days)	Comparing means (SD), HR, adjusted HR using Cox model, rate ratio, Kaplan-Meier estimator	[39,47,52-54]
Adverse events	Reaction to drugs and/or change in health status during course of therapy.	-Interviews	*t*-test	[30,39,51,54]
		-Physical examination		
		-Randomly recorded at visits		
		-Total number of adverse events per day		

### Data abstraction

For the purpose of this review, we will construct full-text extraction forms. Data will be later transferred from these forms and entered into a Microsoft Excel 2011 document. The data abstraction forms were pilot tested in duplicate to ensure their feasibility in this review. These forms are available upon request. Any contention that arises during the extraction process will be resolved through discussion, and if necessary, a third author (ZS) will be brought in. The data extraction forms will allow us to adequately manage the large amount of information being extracted from individual studies. This information includes: title of the journal, number of study participants, study methodology (i.e. RCT and cohort), participant mean age, outcomes assessed, methods of statistical measurement, covariates measured in regression models, outcome statistical association value, *p*-value, confidence intervals, data quality (i.e. percentage of missing data and how missing data were handled), and methods used to correct for multiple testing.

### Statistical analysis plan

When summarizing the evidence of multiple therapies, we often find that there are a limited number of studies providing direct comparisons. For example, a number of systematic reviews compare new therapies (i.e. Naltrexone and heroin-assisted treatment) only to placebo or the standard of care, this being methadone. Using novel statistical approaches to multiple treatment comparisons (MTC) such as the network meta-analysis (NMA), we will provide the pooled effect estimates of all OSTs for continued opioid abuse and patient retention, disseminating both direct and indirect comparisons of all therapies. The results of this review will be summarized both narratively and statistically where possible. For this review, we will provide summary estimates (pooled odds ratios for binary outcomes and standardized mean differences for continuous outcomes) calculated using direct and indirect sources of evidence, as well as those arising from mixing both direct and indirect evidence, provided the assumption of consistency is reasonable.

Due to the stark differences in methodology, we will not be pooling data retrieved from observational studies with data from RCTs. All direct estimates will be pooled separately based on study design (randomized vs non-randomized). While some studies suggest the differences in treatment estimates obtained from well-designed observational research do not differ greatly from RCTs of the same topic [55,56], pooling data from observational studies and RCTs is highly cautioned against [57,58]. This separation stems largely from the inherent differences between RCTs and observational designs, whereby non-randomized designs face high susceptibility to selection bias [57].

#### *Direct comparisons*

Direct evidence will be pooled using a random-effect meta-analysis with Knapp-Hartung (KH) estimator [59]. All analyses will be performed using the metafor and rmeta packages in R [60].

Pooled results from the direct comparisons will be presented in forest plots. The most commonly used estimator is DerSimonian-Laird (DL) and is most often the default estimator in statistical software packages like Review Manager [61]. The DL estimator is demonstrated to be inadequate in capturing study heterogeneity, producing narrow confidence intervals and over-inflating treatment effects [61,62]. The KH estimator works on assumptions that variances are estimated from small samples, in addition to constructing confidence intervals based on the *t*-distribution (with k-1 degrees of freedom) [59,63]. Direct comparisons will weight studies eligible for inclusion using the inverse of the variance. For the direct comparison meta-analyses pooling the results from studies investigating retention in treatment, data will be pooled using risk ratios. The standardized mean difference will be used when pooling the results of studies investigating continued opioid abuse when measured as a continuous variable (mean number of opioid-positive urine screens per treatment arm). Provided we have an appropriate number of studies, we will use an Egger's plot to assess for publication bias.

We anticipate differences in outcome measurement and methodological quality to be important factors for explaining heterogeneity. These differences will be captured in our methodological quality assessment using the modified Newcastle-Ottawa Scale for observational studies and the Cochrane risk of bias tool for RCTs. Scores from these tools are determined from a thorough assessment of study design features such as: sampling strategy, methodological design (e.g. blinding), and outcome measurement (e.g. urine toxicology screening vs self-report). We will conduct subgroup analyses to address the robustness of our results when stratified by methodology quality based on the risk bias assessment scores. Studies will be separated into ‘high and low’ quality based on their scoring, where studies scoring 5 or lower on the Newcastle Ottawa scale and 6 points or higher on the Cochrane risk of bias tool will be assessed. These are standard methodological scoring cut offs used in previous reviews [64]. Provided the data is suitable, we will perform subgroup analyses based on the scoring procedures described above.

Some studies suggest using an I^2^ test statistic cut off of 40% or greater as an indication of heterogeneity among the pooled studies [57]; however, using such thresholds may be ‘misleading’ since heterogeneity represented in the I^2^ statistic is influenced by multiple factors [57]. We will rely on multiple thresholds set forth by the Cochrane Collaboration to aid in our I^2^ statistic interpretation, these include I^2^ of 0%–40% (might not be important), 30%–60% (moderate heterogeneity), 50%–90% (substantial heterogeneity), and 75%–100% (considerable heterogeneity) [57].

#### *Direct and indirect evidence: the network meta-analysis*

We propose using a Bayesian hierarchical model for binary outcomes, where we can account for sampling variability, treatment heterogeneity, and inconsistency while also applying maximum likelihood estimation [65]. The statistical model for the MTC we propose to use allows for an additional random effect, representing change in the treatment effect as a result of the comparison being made [65]. Variation in this random effect across comparisons will be interpreted as inconsistency [65]. Assumptions guiding NMA dictate that trials must be equivalent in their study design and population selection or the statistical results may be compromised [66]; thus, we will only be including evidence from RCTs into the NMA model. When trials evaluating a specific treatment are fundamentally distinct from other trials within that collection, the statistical results may be compromised by inconsistency [66]. To identify inconsistency, we will compare direct and indirect evidence using an approach known as node splitting [67,68]. Comparing inconsistency using this approach allows us to identify the loops with large inconsistency and ultimately consider this during interpretation of the results. Within the Bayesian framework, we will also use the deviance information criterion (DIC) to inform how parsimonious the data are, with a lower value being desired [67].

Provided the data is suitable, we will also address inconsistency using meta-regression to adjust for covariates (effect modifiers) across studies. We will perform a regression using study level data such as OST dose (mg/day), publication date, or study design features (blinding) to examine the improve or change in model fit after covariates are included into the model [67].

We will present our results with probability statements of treatment effects, by which ranking these probabilities allows the advantage of clarifying the sometimes over-complex reporting of pairwise *p*-values [69]. Ranking probabilities allows us to disseminate as a chance percentage, which treatment ranks the highest [69]. After displaying these rank probabilities graphically, we will construct the surface under the cumulative ranking (SUCRA) line for each treatment, in an effort to the graphically displayed probability ranks [69].

#### *GRADE framework*

We will assess the summary estimates of this investigation using the Grading of Recommendations Assessment, Development and Evaluation (GRADE) guidelines [70]. Both direct and indirect estimates obtained from the NMA will be subject to thorough review using the GRADE framework. Evidence from indirect comparisons will be subject to additional scrutiny due to our inability to reliably show that the features of trial design (participants, interventions, outcome measures) are not impacting the observed treatment effect [71].

## Discussion

We anticipate disseminating an objective review of the current available literature on the effectiveness of all substitute opioid therapies for the treatment of opioid use disorder. This review will allow us to evaluate not only the relationship between all substitute opioid therapies and the patient important outcomes, but also, this review will allow us to evaluate the methodological quality of current available evidence. We seek to understand whether there are inconsistencies in the research and what reasons may account for them. Gaining insight into the predictors of patient response characteristics in opioid use disorder will help physicians develop patient-centered treatment regimes. This will be the first systematic review available in the literature looking at all possible substitute opioid therapies at one time. Thus, the dissemination of these results is imperative to the further enhancement of clinical practice through guideline development.

## Abbreviations

OST: Opioid Substitution Treatment; MMT: Methadone Maintenance Therapy; WHO: World Health Organization; HAT: Heroin-assisted therapy; EMBASE: Excerpta Medica DataBase; RCT: Randomized controlled trial; MOOSE: Meta-analysis of Observational Studies in Epidemiology; PRISMA: Preferred Reporting Items for Systematic reviews and Meta-Analyses; NOS: Newcastle-Ottawa Scale.

## Competing interests

The authors declare that they have no competing interests.

## Authors’ contributions

BBD, LN, ZS, and LT conceived the research question and designed the review protocol. BBD and LN completed the initial literature search, developed an electronic search strategy as well as designed and pilot tested the data extraction forms. BBD, AB, and LT developed the direct and multiple treatment comparison statistical analysis plan. BBD, LN, MB, AB, LT, ZS, CP, MV, JD, DCM, DD, GP, and AW contributed equally to writing and revision of the manuscript. The final version of the protocol submitted to BMC Systematic Reviews has been read and approved by all authors. All authors read and approved the final manuscript.
